# Demographic and clinical characteristics of UK military veterans attending a psychological therapies service

**DOI:** 10.1192/pb.bp.113.046474

**Published:** 2014-12

**Authors:** Clarissa M. Giebel, Paul Clarkson, David Challis

**Affiliations:** 1 University of Manchester, UK

## Abstract

**Aims and method** To investigate the demographic and clinical characteristics of subgroups of UK veterans attending a dedicated psychological therapies service following the Improving Access to Psychological Therapies (IAPT) treatment model. Veterans accessing a newly established service in the north-west were categorised into three groups: early service leavers, those with a physical disability, and substance and/or alcohol misusers. Anxiety, depression and social functioning were measured pre- and post-treatment.

**Results** Veterans vary in their demographic and clinical characteristics as well as in treatment efficacy, as measured by the post-treatment scores on probable depression and anxiety. Therapy appears to be most effective in early service leavers, whereas veterans with a physical disability or a substance or alcohol misuse problem tend not to do as well in terms of symptoms of depression or anxiety.

**Clinical implications** This study highlights the importance of targeting different veteran subgroups for dedicated psychological therapy.

Each year, a larger proportion of personnel are leaving the military service in Britain,^1^ which may place great strain on their mental and physical well-being as well as their successful reintegration into society. Although the majority of UK veterans remain fit and well after military service,^2^ returning from war zones into civilian life can precipitate stress or further exacerbate existing mental health problems. To help address this issue, specific services for veterans are available in the UK,^3–6^ including services based on the Improving Access to Psychological Therapies (IAPT) model which aims to improve the availability of psychological treatments for depression and anxiety.^7^ However, evaluations of veteran-specific treatment services are limited and more evidence as to their efficacy is required.

Recent literature testifies to the importance of classifying veterans into specific subgroups, judged to be at greater risk of developing common mental disorders and psychological difficulties, such as: early service leavers (ESL);^8^ reservists;^5^ those who have a physical disability;^9^ and those with substance and/or alcohol misuse.^10^ Recently, ESL have been defined as including veterans who served for more than 4 years, but have committed an offence and been discharged from the military service.^11^ However, data were unavailable on these aspects and therefore we regarded ESL in line with Buckman *et al*’s definition,^8^ whereby veterans are considered to be ESL if they fail to fulfil their contracted service time of between 3 and 4.5 years, depending on their military branch. Compared with non-early service leavers (N-ESL), ESL are reported to have poorer mental health,^8^ which may be the reason for leaving military service early in the first place. Support for this comes from a three-phase cohort study, in which lower levels of psychological well-being and increased stress in phase 1 were associated with leaving the service at phase 3.^2^ Within the same cohort study, a further distinction was made between veterans with and without a physical disability. Consistent with reports on the general veteran population,^12^ 12% of veterans who did not have a physical disability experienced poor mental health, with issues ranging from anxiety to depression and alcohol dependency. In contrast, a larger proportion of veterans with disability (up to 24%) reported lower levels of mental well-being.^9^ Contrary to ESL and veterans with disability, the mental health of reservist veterans has not been explored specifically, but only in combined groups with active reservists.^13,14^ There is, however, a dedicated treatment programme for reservists in place that has been subject to evaluation,^5^ which testifies to the importance of providing psychological treatment directed specifically at this subgroup. Similarly, no study has so far explored the characteristics of those veterans who misuse substances or alcohol and the effect towards treatment, although the importance of this group has been noted.^10^ Considering these subgroups, this study has two aims: first, to assess the demographic characteristics of different groups presenting themselves to a veteran-specific IAPT service; and second, to compare clinical outcomes of anxiety, depression and work and social adjustment between subgroups pre- and post-treatment.

## Method

### Participants

Data were collected as part of a newly initiated IAPT service in the north-west of England targeted at veterans with clinical and social problems. The data included here were collected from patient contacts during September 2011 to April 2013. There were 952 veterans who sought access to the service, of whom 707 (74.3%) were accepted. Of those, 156 (22.1%) completed therapy, 179 (25.3%) remained in therapy at the end of data collection and 289 (40.1%) had dropped out after acceptance or mid-way through therapy. The remainder were awaiting therapy or deemed unsuitable after acceptance (*n* = 83, 11.7%). For the purpose of determining changes in pre- and post-treatment measures among veteran subgroups, clinical measures are only provided for those who attended at least two therapy sessions (*n* = 395), of whom 366 fell into the categories of ESL (or N-ESL), physical disability (or no physical disability) or substance or alcohol misuse (or no misuse). Reservists were not included as a separate subgroup because only 17 accessed the IAPT service, with 10 having received two or more sessions. As opposed to the other subgroups, this number was too small for findings to be generalised.

### Clinical measures

Depressive symptoms were measured using the Patient Health Questionnaire (PHQ-9),^15^ on which scores of 10 or above, out of 27, indicate depression. For anxiety, the Generalised Anxiety Disorder scale (GAD-7)^16^ was employed, whereby a score of 8 or higher out of a total of 21 is indicative of generalised anxiety, post-traumatic stress disorder (PTSD), panic disorder or social anxiety disorder. The 5-item Work and Social Adjustment Scale (WSAS)^17^ assessed social difficulties, with each item being rated between 0 and 8. A cumulative total above 20 or between 10 and 20 indicates marked psychopathology or significant functional impairment respectively.

### Data analysis

Statistical analyses were performed using SPSS version 20 in Windows. Frequency distributions were employed to assess demographic characteristics and clinical measures, including depression, anxiety and work and social adjustment, across subgroups. Independent *t*-tests were used to compare means of clinical measures and chi-squared tests were used to compare distributions of demographic characteristics between subgroups and their counterparts, for example ESL was compared with N-ESL. For all statistical analyses, the levels of significance were reported by *P*-values (*P*<0.05 and *P*<0.01), as indicated in the tables.

Changes in pre- and post-clinical measures between subgroups with two or more sessions were assessed using effect sizes^18^ by subtracting the post-treatment from the pre-treatment mean score and dividing by the post-treatment standard deviation. These provided a standardised indication of impact for the service.

An additional measure of clinical outcome was the ‘caseness’ of anxiety and depression pre- and post-treatment. Caseness status and the proportion of patients who scored above the cut-off points for probable depression or anxiety pre- and post-treatment on the PHQ-9 and GAD-7 were calculated for those with two or more sessions. From this, relative risks of depression or anxiety were calculated as the ‘event rate’ (the numbers with probable depression or anxiety) post-treatment divided by the ‘event rate’ pre-treatment. In this way, the proportion of veterans improving to an extent where symptoms had remitted below the cut-off for depression or anxiety could be discerned.^19^

## Results

When comparing demographic and psychiatric characteristics between those who completed their therapy or remained in treatment against those who dropped out either after acceptance or mid-way through therapy, only forensic history significantly varied (χ^2^ = 4.75, *P*<0.03), with a higher proportion of veterans who dropped out of treatment having had a forensic history (69% as opposed to 43% of veterans who remained in treatment).

### Demographic and psychiatric characteristics of veteran subgroups

Demographic characteristics are displayed in Table 1. In comparing ESL with N-ESL, there were no significant differences in terms of gender, ethnicity, armed forces branch, unemployment status and forensic history. By contrast, relationship status (χ^2^ = 10.95, *P*<0.01) and age (*t*_[134.52]_ = 3.522, *P*<0.01) differed significantly, with ESL tending to be younger and single. With rank on discharge and years of service being partly definitive of ESL status, there was no need to compare these categories. For those with and without a physical disability, age (*t*_[137.45]_ = –5.438, *P*<0.01) and ethnicity (χ^2^ = 5.2, *P*<0.05) differed significantly between groups, with veterans referred with a physical disability tending to be older and from different ethnic backgrounds. For those with and without substance or alcohol misuse, age (*t*_[400.13]_ = 3.768, *P*<0.01), gender (χ^2^ = 8.87, *P*<0.05), relationship status (χ^2^ = 31.87, *P*<0.01), rank on discharge (χ^2^ = 21.64, *P*<0.05), years of service (*t*_[403.61]_ = 3.919, *P*<0.01), unemployment (χ^2^ = 8.13, *P*<0.05) and forensic history (χ^2^ = 46.02, *P*<0.01) differed significantly. Veterans who had evidence of substance misuse and were referred to the service tended to be younger, male, single or divorced privates with fewer years of service and greater levels of unemployment and forensic history.

With respect to mental health status, the most common psychiatric problem among the total sample was PTSD, followed by mixed depression and anxiety and by depression. Only a small percentage experienced stand-alone anxiety (6%), with other disorders including bipolar affective disorder, obsessive–compulsive disorder and mental disorders caused by alcohol misuse. The distribution of psychiatric disorders varied significantly between those with and without

**Table 1:** Demographic characteristics of all veterans who presented to the service

	ESL (*n* = 107)	N-ESL (*n* = 568)	Physical disability (*n* = 77)	No disability (*n* = 398)	Substance/ alcohol misuse (*n* = 168)	No misuse (*n* = 323)	Total (*n* = 952)
Age, years: mean (s.d.)	39 (13.4)	44 (11.1)	48 (8.7)	42 (11.9)	41 (9.9)	44 (12)	43 (11.7)
							
Gender, *n* (%)^a^							
Female	9 (8.4)	32 (5.6)	5 (6.5)	31 (7.8)	4 (2.4)	27 (8.4)	75 (7.9)
Male	98 (91.6)	536 (94.4)	72 (93.5)	367 (92.2)	164 (97.6)	296 (91.6)	877 (92.1)
							
Ethnicity, *n* (%)^a^							
White British	73 (98.6)	426 (96.8)	65 (91.5)	373 (97.1)	154 (96.3)	303 (96.2)	590 (96.7)
Other	1 (1.4)	14 (3.2)	6 (8.5)	11 (2.9)	6 (3.7)	12 (3.8)	20 (3.4)
							
Relationship status, *n* (%)^a^							
Married^b^	17 (23)	171 (40.9)	30 (42.3)	144 (37.9)	35 (21.5)	150 (47.6)	213 (38.1)
Divorced^c^	16 (21.6)	94 (22.5)	15 (21.1)	83 (21.8)	44 (27)	61 (19.4)	124 (22.2)
Single	41 (55.4)	153 (36.6)	26 (36.6)	153 (40.3)	84 (51.5)	104 (33)	222 (39.7)
							
Branch of force, *n* (%)^a^							
Army	96 (90.6)	489 (86.7)	63 (82.9)	344 (87.6)	148 (88.1)	278 (86.3)	774 (87.5)
Navy/Marines	4 (3.8)	37 (6.6)	6 (7.9)	26 (6.7)	9 (5.4)	24 (7.5)	55 (6.2)
Royal Air Force	5 (4.7)	38 (6.7)	7 (9.2)	22 (5.6)	10 (6)	8 (2.5)	53 (6)
							
Rank on discharge, *n* (%)^a^							
Private	61 (91)	241 (60.1)	46 (69.7)	224 (63.6)	117 (74.5)	176 (59.5)	332 (64.6)
JNCO	6 (9)	99 (24.7)	13 (19.7)	82 (23.3)	29 (18.5)	75 (25.3)	116 (22.6)
SNCO	-	50 (12.5)	5 (7.6)	37 (10.5)	9 (5.7)	36 (12.2)	54 (10.5)
Officer	-	11 (2.7)	2 (3.0)	9 (2.6)	2 (1.3)	9 (3)	11 (2.1)
							
Years of service, mean (s.d.)	2.6 (2.2)	9.6 (6.1)	8.8 (5.6)	8.8 (6.8)	7.3 (5.1)	9.5 (6.9)	8.4 (6.1)
							
Unemployment, *n* (%)^a^	12 (21.8)	109 (27.9)	20 (31.7)	85 (24.6)	46 (31.9)	67 (23.1)	139 (27.7)
							
Forensic history, *n* (%)^a^	24 (39.3)	123 (32.9)	16 (25)	113 (34.5)	83 (55.3)	71 (23.7)	161 (34.8)
							
Psychiatric disorder, *n* (%)^a^							
Depression	24 (30.8)	112 (24.2)	17 (23.6)	106 (28.6)	38 (24.4)	84 (27.7)	165 (24.1)
Anxiety	7 (9)	25 (5.3)	4 (5.6)	25 (6.8)	7 (4.5)	22 (7.3)	43 (6.3)
Mixed A&D	22 (28.2)	134 (29)	12 (16.7)	111 (30)	45 (28.8)	86 (28.4)	197 (28.8)
PTSD	19 (24.4)	163 (35.3)	37 (51.4)	108 (29.2)	54 (34.6)	99 (32.7)	233 (34)
Other	6 (7.7)	28 (5.1)	2 (2.8)	20 (5.4)	12 (9.6)	12 (3.9)	47 (6.8)

A&D, anxiety and depression; ESL, early service leavers; JNCO, Junior Non-Commissioned Officer; N-ESL, non-early service leavers; PTSD, post-traumatic stress disorder; SNCO, Senior Non-Commissioned Officer.

a. Percentages are valid per cent.

b. Married or in a civil partnership.

c. Divorced, dissolved civil partnership, separated or widowed.

substance or alcohol misuse issues (χ^2^ = 246.241, *P*<0.01) and between those with and without a physical disability (χ^2^ = 14.509, *P*<0.05), albeit for the latter, two cells contained fewer than five cases. Mental health problems were not markedly differently distributed between ESL and N-ESL.

### Clinical outcomes for veteran subgroups with two or more sessions

In Table 2, means, standard deviations and effect sizes of change from clinical measures pre- and post-treatment are displayed. Veterans with a physical disability tended to show the highest average scores in all three domains, suggesting worse symptomatology and adjustment, compared with all other subgroups. With respect to ESL and N-ESL, only post-WSAS scores (*t*_[331]_ = 2.253, *P*<0.05) differed significantly. However, the standardised effect size showed that there was a greater impact of the service if the patient was an ESL. For the physical disability subgroup, pre-PHQ (*t*_[82.49]_ = –2.997, *P*<0.01), pre-GAD (*t*_[307]_ = –2.571, *P*<0.05) and post-GAD (*t*_[308]_ = –2.127, *P*<0.05) as well as pre-WSAS (*t*_[305]_ = –4.154, *P*<0.01) and post-WSAS scores (*t*_[305]_ = –3.674, *P*<0.01) differed significantly from the scores for veterans without a disability. Veterans with a disability also reported higher levels of anxiety and depression both pre- and post-treatment, albeit with a greater improvement in depression (as indicated by the effect size). Improvement on symptoms of anxiety showed no large variations between the two subgroups. For substance and alcohol misusers, their post-PHQ (*t*_[339]_ = 2.125, *P*<0.05), pre-GAD (*t*_[413]_ = 2.031, *P*<0.05) and post-GAD score (*t*_[339]_ = 2.106, *P*<0.05) differed significantly from scores of veterans without substance or alcohol misuse. This is supported by slightly higher effect sizes for the latter group, which indicates that treatment was more efficacious for these veterans as opposed to alcohol or substance misusers. These measures indicate that membership of a subgroup predicts different treatment effects from non-membership, particularly for physical disability. Overall, the largest effect sizes and thus the greatest improvement through treatment are reported for ESL across all three domains of anxiety, depression and work and social adjustment.

**Table 2:** Clinical measures pre- and post-treatment for each subgroup with two or more sessions

	Mean (s.d.)						
	ESL (*n* = 43)	N-ESL (*n* = 296)	Physical disability (*n* = 56)	No disability (*n* = 257)	Substance/ alcohol misuse (*n* = 109)	No misuse (*n* = 232)	Total (*n* = 366)
PHQ-9							
Pre	17.1 (6.5)	17.2 (6.1)	18.9 (5.3)	16.5 (6.2)	17.9 (5.7)	16.8 (6.4)	17 (6.3)
Post	10.2 (8.3)	12.2 (8)	13.1 (8)	11.3 (7.8)	13.2 (8)	11.3 (7.9)	12 (8)
Effect size	0.83	0.63	0.73	0.54	0.59	0.7	0.63
							
GAD-7							
Pre	14.3 (5.3)	14.9 (5.2)	16.1 (4.7)	14.2 (5.2)	15.3 (5)	14.5 (5.2)	14.6 (5.3)
Post	8.8 (7)	10.5 (6.7)	11.8 (6.6)	9.6 (6.7)	11.4 (6.8)	9.7 (6.7)	10.3 (6.8)
Effect size	0.79	0.66	0.65	0.69	0.57	0.72	0.63
							
WSAS							
Pre	21.3 (11.7)	21.6 (10.3)	26.5 (9.2)	20.3 (10.3)	22.4 (10.3)	21.3 (10.6)	21.3 (10.6)
Post	12.5 (11.9)	17 (12)	21.4 (12.7)	14.9 (11.5)	17.1 (11.8)	16.2 (12.2)	16.5 (12)
Effect size	0.74	0.38	0.4	0.47	0.45	0.42	0.4

ESL, early service leavers; GAD-7, Generalised Anxiety Disorder scale; N-ESL, non-early service leavers; PHQ-9, Patient Health Questionnaire; WSAS, Work and Social Adjustment Scale.

Table 3 shows the prevalence of clinical cases of depression and anxiety as well as the relative risk of anxiety and depression across the different subgroups and across the total sample. There was a suggestion that therapy was effective across all subgroups in reducing the number of veterans that had depression or anxiety. Although the proportion of veterans within each subgroup displaying symptoms of depression or anxiety is similar at between 85 and 94%, the highest levels of caseness post-treatment were found for those with a physical disability and those misusing alcohol or substances compared with other groups. Veterans not belonging to any subgroup had the lowest proportion of probable cases of depression or anxiety post-treatment, whereby fewer ESL reported symptoms of anxiety or depression above the respective cut-offs, with ESL having remitted to the greatest extent.

## Discussion

This study compared the demographics and mental health outcomes of different veteran subgroups, perceived to be at risk of depression or anxiety, accessing a veteran-specific psychological therapies service. Data were collected from

**Table 3:** Clinical cases of depression and anxiety pre- and post-treatment for subgroups with two or more sessions

	*n* (%)						
	ESL (*n* = 43)	N-ESL (*n* = 296)	Physical disability (*n* = 56)	No disability (*n* = 257)	Substance/ alcohol misuse (*n* = 109)	No misuse (*n* = 232)	Total (*n* = 366)
Depression							
Pre-treatment	38 (88.4)	259 (87.5)	50 (94.3)	223 (86.8)	97 (89)	202 (87.1)	316 (86.3)
Post-treatment	22 (51.2)	173 (58.6)	34 (64.2)	142 (55.3)	66 (61.7)	129 (55.1)	213 (58.2)
Relative risk	0.58	0.67	0.68	0.64	0.68	0.64	0.67
							
Anxiety							
Pre-treatment	39 (90.7)	256 (86.8)	47 (88.7)	223 (86.8)	99 (90.8)	200 (86.2)	314 (85.8)
Post-treatment	22 (51.2)	183 (62)	36 (67.9)	149 (58)	74 (69.2)	132 (56.4)	223 (60.9)
Relative risk	0.56	0.71	0.77	0.67	0.74	0.66	0.71

ESL, early service leavers; N-ESL, non-early service leavers.

the first set of patients who entered a newly established IAPT service for veterans in the UK, and variations between ESL, veterans with a physical disability and those with alcohol or substance misuse issues were compared. Only recently have specific veteran subgroups been studied, with only a very sparse evidence base existing on the relationship between substance and alcohol misuse and mental health among veterans.^20^ Differences in demographics and clinical outcomes are discussed below.

Examining variations in psychiatric disorders, the high frequency of PTSD, depression and anxiety among all referred veterans were in line with other reports on veteran mental health.^6,21^ Interestingly, diagnoses varied significantly between those with and without substance or alcohol misuse and those with and without a physical disability. Concerning the former, substance or alcohol misuse is not only found concurrently with mental illnesses^22^ but is also likely in some cases to lead to poor psychological well-being.^23,24^ Hence, mental health problems in veterans with substance or alcohol problems are directly linked to their membership of that particular subgroup, whereas veterans lacking these have mental health problems more dependent on other factors. Similarly, veterans with a physical disability showed variations in diagnoses compared with veterans without disability. In particular, more than half of veterans with disability had a diagnosis of PTSD, reflecting previous literature noting the link between disability and PTSD symptoms.^25^ Identifying such variations in psychiatric diagnoses between subgroups can prove of importance in clinical practice in shaping therapeutic methods, such as a greater focus on PTSD in veterans with a disability.

Turning to clinical outcomes for different subgroups, it emerged that symptoms of depression, anxiety and work and social adjustment varied pre- and post-treatment, particularly between those who had and those who did not have a disability. Changes in work and social adjustment differed particularly between all three subgroups, with substance or alcohol misusers and veterans with a disability reporting the poorest adjustments to their work and social life both pre- and post-treatment. As the WSAS enquires about the effect of a disorder on the ability to perform a job and activities of daily living, social leisure activities as well as maintenance of close relationships,^17^ these effects may refer to the physical disability or addiction as the causal factor, rather than to mental health problems of depression or anxiety. Physical disability could be associated with difficulties in performing certain types of jobs and tasks at home, and it may also reduce the possibility of taking part in certain leisure activities. Similarly, substance or alcohol addiction can have a negative impact on holding down a job and maintaining stable relationships with family and friends. This may explain the remaining poor, albeit improved, outcome on this aspect of therapy.

Focusing on recovery from depression and anxiety, variations are only evident post-treatment and thus in the efficacy of treatment on individual subgroups. Contrary to veterans with a physical disability or alcohol or substance misuse, who have the highest post-treatment risks of anxiety and depression, indicating the lowest treatment efficacy, ESL have the overall lowest risk of having either disorder. This indicates that treatment is most effective for ESL. Considering that ESL tend to be younger than N-ESL and leave the service significantly earlier, it is possible they are exposed to traumatic situations that may contribute to depression or anxiety for a shorter period of time. Although ESL show similar levels of caseness prior to treatment, they may recover slightly better due to the shorter exposure to stressful situations than other veterans. With regard to the high risk of anxiety and depression in the other two subgroups, it may be that the additional impact of physical disability contributes to the reduced efficacy of treatment. This is supported by literature indicating the positive association between reduced mobility – and thus increased dependence – and anxiety and depression.^26,27^ On the other hand, substance and alcohol misuse are also linked to heightened levels of depression and anxiety, whereas substance misuse concurrent with depression has been associated with less effective treatment, similar to the present findings.^28,29^ Consequently, different subgroups would seem to require special attention within therapy, as membership of each subgroup can shape treatment efficacy.

### Limitations

This study has investigated the differential impact of veteran subtypes on treatment outcomes and service engagement. However, some factors limit the assessment of veteran subgroups and interpretation of effects. One issue is the small size of groups with a particular characteristic compared with those without the characteristic. For this reason, reservists were not evaluated separately as their number was insufficient to permit generalisation. However, ESL, physical disability and substance or alcohol misuse samples were large enough to provide reliable information about their demographics and clinical measures. The second limitation was missing data, with not all demographic information being recorded for each assessed veteran. Consequently, the sample may have contained more ESL and veterans with physical disability or substance or alcohol misuse who were not included in the analyses. Third, it needs to be noted that subgroups are not mutually exclusive, so that ESL may also fall into the category of physical disability or substance or alcohol misuse. This may create a more complex profile of individual veterans, which would lead to clinicians having to address not only mental health needs, but also the combined effect of several veteran characteristics that are shown to mediate treatment efficacy. A fourth possible limitation may be aetiology of problems. The Medical Assessment Programme (MAP) found that some 10% of attendees had either exaggerated their experiences or not had them at all.^6^ Unfortunately, such information was not available to us from the National Health Service trust as part of their routine data collection, so we could not investigate this issue.

### Implications

The findings of this study have implications for both research and clinical practice and add to the sparse evidence base on veteran characteristics in relation to mental health. First, understanding the differential effects of demographics, personal history and previous mental health on clinical outcomes can help to discriminate different response patterns according to these variations. Rather, services and therapists should address each group or combination of characteristics with special attention, as this may increase treatment efficacy. Such more targeted therapy, as opposed to a more generic response, may enhance treatment, which could in turn reduce the number of required sessions and thus the costs associated with treatment.
